# Static self-directed sample dispensing into a series of reaction wells on a microfluidic card for parallel genetic detection of microbial pathogens

**DOI:** 10.1007/s10544-015-9994-1

**Published:** 2015-08-11

**Authors:** Robert D. Stedtfeld, Yen-Cheng Liu, Tiffany M. Stedtfeld, Tanja Kostic, Maggie Kronlein, Onnop Srivannavit, Walid T. Khalife, James M. Tiedje, Erdogan Gulari, Mary Hughes, Brett Etchebarne, Syed A. Hashsham

**Affiliations:** Civil and Environmental Engineering, Michigan State University, East Lansing, MI 48824 USA; The Center for Microbial Ecology, Michigan State University, East Lansing, MI 48824 USA; Department of Microbiology and Molecular Genetics, Michigan State University, East Lansing, MI 48824 USA; Department of Chemical Engineering, University of Michigan, Ann Arbor, MI 48109 USA; Department of Microbiology, Sparrow Laboratories, Sparrow Health System, Lansing, MI 48912 USA; Department of Osteopathic Medical Specialties, Section of Emergency Medicine, College of Osteopathic Medicine, Michigan State University, East Lansing, MI 48824 USA; Bioresources Unit, AIT Austrian Institute of Technology GmbH, Konrad Lorenz Strasse 24, A-3430 Tulln, Austria

**Keywords:** Static self-directed sample dispensing, Airlock mechanism, Laser etched, Disposable microfluidic card, Isothermal amplification, POC, Genetic diagnostics, Gene-Z, LAMP

## Abstract

A microfluidic card is described for simultaneous and rapid genetic detection of multiple microbial pathogens. The hydrophobic surface of native acrylic and a novel microfluidic mechanism termed “airlock” were used to dispense sample into a series of 64 reaction wells without the use of valves, external pumping peripherals, multiple layers, or vacuum assistance. This airlock mechanism was tested with dilutions of whole human blood, saliva, and urine, along with mock samples of varying viscosities and surface tensions. Samples spiked with genomic DNA (gDNA) or crude lysates from clinical bacterial isolates were tested with loop mediated isothermal amplification assays (LAMP) designed to target virulence and antibiotic resistance genes. Reactions were monitored in real time using the Gene-Z, which is a portable smartphone-driven system. Samples loaded correctly into the microfluidic card in 99.3 % of instances. Amplification results confirmed no carryover of pre-dispensed primer between wells during sample loading, and no observable diffusion between adjacent wells during the 60 to 90 min isothermal reaction. Sensitivity was comparable between LAMP reactions tested within the microfluidic card and in conventional vials. Tests demonstrate that the airlock card works with various sample types, manufacturing techniques, and can potentially be used in many point-of-care diagnostics applications.

**Electronic supplementary material:**

The online version of this article (doi:10.1007/s10544-015-9994-1) contains supplementary material, which is available to authorized users.

## Introduction

Estimates of mortality caused by infectious diseases range from 19 to 25 % worldwide (Fauci and Morens 2012; Lozano et al. 2010). Point of care (POC) diagnostics that are affordable, sensitive, specific, simple, equipment-less, rapid, and robust (Mabey et al. 2004) are expected to reduce time to intervention and influence positive outcomes. Microfluidic chips/cards with the capacity to target multiple infectious diseases in parallel are a promising means to rapidly diagnose and satisfy many POC criteria (McCalla and Tripathi 2011). Innovative strategies for precise microfluidic control or loading and sealing samples into multiple well compartments (for parallel detection) have been described and reviewed (Jung et al. 2015; Sackmann et al. 2014; Su et al. 2014) including: the manual deposition of sample in a small number of individual wells (Fang et al. 2010; Lee et al. 2008), the utilization of peripheral equipment to load samples with arrayers and injectors (Matsubara et al. 2004; Morrison et al. 2006; Duarte et al. 2013), pumps (Furuberg et al. 2008; Sun et al. 2015), centrifuges (Lutz et al. 2010; Jung et al. 2014; Hoehl et al. 2014), development of multiple-layer cards utilizing slip (Shen et al. 2011), air, thermal, magnets, physical valve control (Fang et al. 2012; Stedtfeld et al. 2012; Trung et al. 2010; Wang et al. 2011), vacuum assistance (Abe et al. 2011), or immiscible fluids to prime cards (Gansen et al. 2012), surface treatment for primer immobilization and mobilization during filling (Morrison et al. 2006), and finally, propagation of sample through a parallel network of interconnecting channels using an air vent for each reaction well (Fang et al. 2010; Ramalingam et al. 2010; Stedtfeld et al. 2012; Tourlousse et al. 2012). However, most microfluidic cards made of glass, silicon, and polymers require highly sophisticated microfabrication (e.g. multiple layers and surface functionalization), peripheral instruments, or complicated liquid handling that do not satisfy POC criteria (Nilghaz et al. 2012; Hu et al. 2014). Without further reduction in cost or operational simplicity, the value and adaptability of microfluidic devices for POC genetic diagnostics is diminished (Su et al. 2014; Volpatti and Yetisen 2014).

To better meet POC criteria, our goal was to develop disposable cards with 1) a single-etched layer that does not require fine alignment, clean rooms, or surface functionalization, and 2) distributes sample into a series of reaction wells without carryover of dried pre-existing reaction constitutes, without the need for external peripherals, extended loading times, or numerous open points increasing the likelihood for contamination and evaporation. Unique processes utilized in the development of the card include a microfluidic network that traps air on one side of a bifurcated channel forming a plug termed “airlock,” and static self-directed sample dispensing into larger volume wells due to the hydrophobic nature of native acrylic. These mechanisms prevent carryover of dried constitutes into subsequent wells, and complete loading of sample into reaction wells preceding further flow, respectively.

Cards were tested for proper loading with dilutions of blood, urine, saliva, and mock samples prepared with varying viscosities and surface tensions. The multi-well card was also tested for parallel detection of multiple genetic markers related to identification, virulence, and antibiotic resistance from clinical pathogens. Loop mediated isothermal amplification (LAMP) reactions were performed with mixtures of genomic DNA (gDNA) and crudely lysed body fluid samples spiked with bacterial cells. LAMP reactions were monitored in real time using a previously described inexpensive and compact device termed Gene-Z (Stedtfeld et al. 2012). Endpoint images of the amplification reaction within the card were also captured using a CCD.

## Material and methods

### Microfluidic card fabrication

Cards were designed using Argon CAD 3D software (Ashlar-Vellum), converted to AutoCAD DXF (Drawing Interchange Format), and imported into InkScape (Software Freedom Conservancy, Inc.). InkScape was used as an intermediate software between DXF files and RetinaEngrave 3D, which was used to control the desktop CO_2_ Laser (MLE-40, Full Spectrum Laser LLC). Cards were fabricated from 1.59 mm acrylic sheets (McMaster-Carr). The cutting power and speed of the laser were programmed and controlled via RetinaEngrave 3D. The reaction wells were fabricated by engraving with 45 % power and 20 % speed; and the micro-channels were cut with 30 % current, 20 % speed and various power settings (5-65 %) to obtain desired channel depths.

Three different configurations were designed in this study: a card in which one inlet distributes samples into all 64 wells, a card with two inlets each leading to 32 wells, and a card with four inlets each leading to 16 reaction wells (described as one lane herein). The spatial area of the 64 well card was 75 × 58 mm. The liquid channel was approximately 250 μm wide × 250 μm deep (cut with 7 % power) and air segments were 200 μm wide × 200 μm deep (cut with 5 % power). Channels were etched with dimensions to permit fabrication outside of a clean room and prevent clogging with dust particles (i.e. channels dimension larger than 150 μm). The oval-shaped reaction wells were 1400 × 2000 μm long axis × 1000 μm deep engraved with 45 % power, resulting in a volume of ~1.8 μL. The total sample volume was 160 μL. Wells and dimensions of the card were measured using a digital microscope (Model #44,302-A) and a contact surface profilometer (NanoMap-500LS). Inlet and outlets that fit a 200 μL pipette tip (used for filling the cards) were fabricated by laser etching 0.6 μm circles with 30 % power. The time required for engraving a single 64-well card was approximately 10 min. With optimization of laser power and speed, this time may be reduced to less than 5 min.

After laser micromachining, the card was rinsed with distilled water, scrubbed with a paper towel, and rinsed with 70 % ethanol. This cleaning procedure was performed to remove acrylic residues, which was essential for correct flow of sample and complete filling of reaction wells. Cleaned cards were dried on a 70^o^C heater for 5 min. Once dry, compressed air was sprayed over the cards to remove dust, and deposits from the paper towel. For cards tested with LAMP, primers or gDNA were dispensed into wells and dehydrated on a bench-top heater at 70 °C for 5 min. Next, engraved channels and wells were enclosed via a biocompatible optical film with pressure-sensitive silicon adhesive (MicroAmp Optical Adhesive Film; Applied Biosystems, Carlsbad, CA, USA). Secure bonding of the film to acrylic was ensured using a press (4386; Carver, Wabash, IN, USA) at 3000 lb of pressure sandwiched between rubber sheets (McMaster Carr). It should be noted that all other LAMP constitutes (with the exception of primers predispensed into the card) were mixed with sample and loaded into the card at the time of testing.

A screw cap, cut from the top of a 1.5 mL cryogenic vial (TS Scientific, Perkasie, PA, USA) was fixed on the card with UV glue (Dymax Corporation, Torrington, CT, USA) and 5 min of exposure to UV light (PSD Series, Novascan). To ensure sealing, a 0.3 mL piece of polyester wax (melting point of 37 °C, Electron Microscopy Sciences, Hatfield, PA, USA) was fixed to the top of the screw cap. When the LAMP reaction was performed at 63 °C, the wax melted and covered the inlet/outlet holes. Using wax to prevent contamination during the LAMP reaction was reported earlier by Tao and co-authors (Tao et al. 2011). The screw caps encompassed both loading and air release ports. Cards were stored for no more than 1 week prior to use.

Prior to loading, sterile needles were used to pierce the film above the loading port, and sample was dispensed using a 200 μL micropipette (GPS-L250, Rainin). Pressure exerted by the pipette distributed sample into the channels and reaction wells. In this process, air inside channels was purged through a single air vent placed downstream from the series of reaction wells.

### Primer design and loop-mediated isothermal amplification with gDNA

LAMP primers targeting virulence and antibiotic resistance markers from bacterial pathogens were designed using PrimerExplorer4 or retrieved from the literature (Table S1). Genomic DNA (gDNA) from *Salmonella enterica* (ATCC 19585, 13311), *Clostridium difficile* (ATCC BAA-1382D) *Vibrio cholerae* (ATCC 39315), *Campylobacter jejuni* (ATCC 700819), *Giardia intestinalis* (ATCC 30888), *Staphylococcus aureus* (ATCC 700699D), *Streptococcus. agalactiae* (ATCC BAA-1138), *Enterococcus faecalis* (ATCC 19433), *Yersinia enterocolitica* (ATCC 55075), and *Eschericia coli* (ATCC BAA-460D, PTA-5184) were obtained from the American Type Culture Collection (Manassas, VA). The LAMP primers were predispensed in the cards prior to chip assembly, to result in a final concentration of 1.6 μM each of FIP and BIP primers, 800 nM each of LF and LB primers, and 200 nM each of F3 and B3 primers. After cards had been enclosed with the optical film, LAMP reaction mixtures consisting of 800 mM betaine (Sigma Aldrich), 1.4 mM of each dNTP (Invitrogen), 20 mM Tris-HCl (pH 8.8), 10 mM (NH_4_)_2_SO_4_, 10 mM KCl, 8 mM MgSO_4_, 8 mM Triton X-100, 0.64 units/μL of Bst polymerase (New England Biolabs), 20 μM of SYTO 81 dye (Invitrogen), and varying amounts of template with gDNA, crude lysed, or lysates from bacterial isolates were used (as described below). Results are described as genomic copies per reaction for samples spiked with gDNA and CFU per reaction for samples spiked with crude lysates.

For the experiment testing diffusion and carryover of gDNA predispensed in cards, template DNA from ten different organisms was used including *Salmonella 13311* (Sal1) *Salmonella 19585* (Sal2), *E. coli* PTA-5184 (EC1), *E. coli* BAA-460D (EC2), *Y. enterocolitica* (YE), *C. difficile* (CD), *G. intestinalis* (GI), *V. cholera* (VC), *E. faecalis* (EF) and *C. jejuni* (CJ). Two wells were no loaded with template DNA (none).

For all amplification experiments performed in vials, primers were added into reaction mixture to have the same concentrations used in the microfluidic cards.

### Blood, urine, saliva and LAMP reactions dispensed into microfluidic card

Saliva, blood, and urine samples were spiked with dilutions of bacterial isolates collected at Sparrow hospital, Department of Microbiology, in Lansing, Michigan. *Staphylococcus aureus*, *Eschericia coli*, Group A *Streptococcus pyogenes* (GAS), and Group B *Streptococcus agalactiae* (GBS) were isolated using standard culture techniques in the Sparrow Clinical Microbiology Laboratory. Culture identification was performed using Siemens Microscan, BD Phoenix, or biochemical tests. Prior to revival, isolates were stored in 15 % glycerol stocks at −80 °C. Isolates were revived by growing on TSB media overnight at 37 °C (no agitation) and serial diluted in 1 × PBS. Ten microliters of each serial dilution was plated on TSA plates (in triplicate) and colony forming units were counted following 24 h of incubation at 37 °C. Genomic DNA was also extracted from 1.5 mL of the revived stocks using the Qiagen Blood and Tissue kit, following the protocol for Gram-positive bacteria.

Initial experiments were performed with gDNA from the isolates to verify specificity of primer sets and ensure identity of bacterial isolates. These initial experiments were performed in the commercially available real time thermal cycler (Chromo4^™^ PCR thermal cycler, BioRad) under isothermal conditions. Tests included duplicate replicates for each reaction, positive controls with a type strain and no template controls. Type strains were spiked to have a total mass of 1 ng per reaction, and 2 μL was added from isolate lysates yielding 472, 24, 22, and 53 ng/reaction for *S. aureus*, GAS, GBS, and *E. coli*, respectively (Table S2). Specificity of LAMP assays and concordance with culture based identification was tested using 23 LAMP assays against 11 re-cultured isolates derived from septic patients (Table S3).

Whole blood was collected from a healthy volunteer into standard hospital purple top specimen collection EDTA tubes. Whole-blood samples were stored at 4 °C for up to 60 days prior to use. Urine and saliva samples were collected from healthy volunteers in Eppendorf vials, and were stored at 4 °C for up to 14 days prior to use. Serial dilutions of isolates were spiked into blood, urine, and saliva samples. Bacterial content of dilutions was counted following 24 h of growth on TSA plates, as described above, and prepared dilutions are listed as CFU/reaction well (Table 1). Blood sample were spiked with *E. coli* and *S. aureus* isolates, saliva sample were spiked with Group A Streptococcus (GAS), and the urine samples were spiked with Group B Streptococcus (GBS). After spiking with isolates, saliva and urine samples were heat lysed at 95 °C for 5 min. Blood samples were tested via LAMP amplification in the microfluidic card directly without lysing. It should be noted that heat lysed blood samples clotted, and could not be handled with a micropipette. Blood samples were mixed at 1, 5, 10, and 40 % of the total LAMP reaction volume. Urine samples were tested by mixing in LAMP reactions to be 10, 25, and 40 % of total reaction volume, and saliva was mixed to be 10 and 25 % of total reaction. Cards were prepared with 16 reaction wells per sample, and thus four separate samples were loaded per card. With this configuration, three serial dilutions of bacterial isolates and a non-spiked (no template) control was loaded on each card. Cards were preloaded with primers, as described above, with each primer was dispensed into three individual reaction wells. Genomic DNA from a plant pathogen was spiked as an internal positive control into all blood, saliva, and urine samples for a final concentration of 5.7 pg/μL (~60 copies/reaction) to test for inhibition in samples. A primer set targeting the positive control was also loaded into one reaction well of each 16 well lane for cards tested with blood, and four reaction wells out of each 16 well lane for cards tested with GBS and GAS.

**Table 1:** Experimental summary and sensitivity of assays. Positive amplification calls “+” were based on 2 or more of 3 replicate wells displaying increased signal with Gene-Z and CCD images

SAMPLE MATRICES (CARD REPLICATES) CFU/REACTION	Proper well loading	Pos. control	stx2	uidA	coa	mecA	nuc
40 % blood (2)	125/128						
No spike control		+^a^	−	−	−	−	−
460 Ec, 320 Sa		+^a^	−	−	−	−	+^#^
4600 Ec, 3200 Sa		+^a^	−	+^a^	−	−	+^a^
46,000 Ec, 32,000 Sa		+^a^	−	+^a^	+^a^	−	+^a^
10 % blood (3)	192/192						
No spike control		+^a^	−	−	−	−	−
115 Ec, 80 Sa		+^a^	−	−	−	−	−
1150 Ec, 8000 Sa		+^a^	−	+^a^	+^a^	−	+^a^
11,500 Ec, 800 Sa		+^a^	−	+^a^	−	−	+^a^
5 % blood (2)	128/128						
No spike control		+	−	−	−	−	−
57 Ec, 40 Sa		+	−	−	−	−	+
575 Ec, 4000 Sa		+	−	+	+	−	+
5750 Ec, 400 Sa		+	−	+	−	−	+
1 % blood (3)	190/192						
No spike control		+	−	−	−	−	−
11 Ec, 8 Sa		+	−	−	−	−	+
115 Ec, 800 Sa		+	−	+	+	−	+
1150 Ec, 80 Sa		+	−	+	−	−	+
SAMPLE MATRICES (CARD REPLICATES) CFU/REACTION		Pos. control	lmb	mstA		cfb	scpA
40 % Urine (1)	64/64						
No spike control		−	−	−		−	−
12 GBS		−	−	−		−	−
125 GBS		−	−	−		−	−
1250 GBS		−	−	−		−	−
25 % Urine (2)	126/128						
No spike control		+	+	−		−	−
7.5 GBS		+	+	−		+	+
75 GBS		+	+	−		+	+
750 GBS		+	+	−		+	+
10 % Urine (3)	192/192						
No spike control		+	+	−		−	−
3.1 GBS		+	+	−		+	+
31 GBS		+	+	−		+	+
315 GBS		+	+	−		+	+
25 % Saliva (2)	125/128						
No spike control		+	−	−		−	−
0.5 GAS		+	+	−		−	+
5.0 GAS		+	+	−		−	+
50 GAS		+	+	+		−	+
10 % Saliva (3)	190/192						
No spike control		+	−	−		−	−
0.2 GAS		+	+	−		−	+
2.0 GAS		+	+	−		−	+
20 GAS		+	+	−		−	+
Lab prepared Fluid Viscosity (0.69, 0.89, 1.01, 1.40, and 1.80 mPa*s)	256/256						
Lab prepared Fluid Surface Tension (50, 60, and 70 mN/m)	192/192						
Total (28)	1780/1792	99.3 %					

*Abbreviations* are *Ec E.coli*, *Sa S. aurues*, *GBS* Group B Streptococcus, *GAS* Group B Streptococcus

^a^Endpoint images captured with CCD, amplification curves not measurable on Gene-Z

### Ethics statement

All experimentation with human samples was conducted following the Human Research Protection Program and performed in accordance with institutional regulations after pertinent review and approval by the Institutional Review Board at Michigan State University, East Lansing, MI. Michigan State University and Sparrow Hospital Investigational Review Board human subjects exemption with MSU IRB# 12-706; i041390 determined to be non-human subject research. Written consent was obtained from healthy individuals that donated blood, urine, and saliva samples. Cultured bacterial isolates used for spikes were determined to be non-human subject research.

### LAMP monitored with Gene-Z

**Fig. 1:**
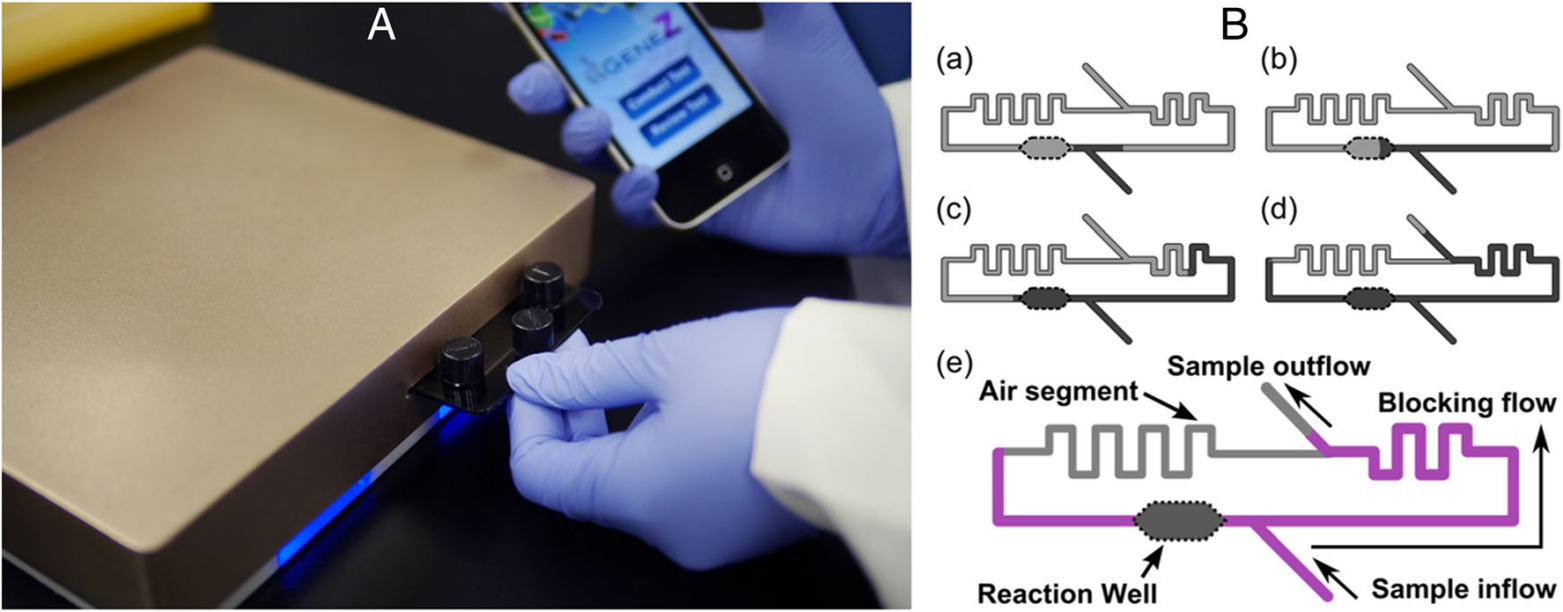
Picture of the Gene-Z prototype (**A**), with airlock card inserted/removed through an opening on the side of the device. (**B**) Schematic diagram of airlock mechanism. (*a*) Liquid bifurcates into two channels. (*b*) Liquid fills the left channel with the reaction well. (*c*) After the reaction well fills, liquid continues in both the right and left channels. (*d*) The channel downstream from the well does not fully fill as liquid is blocked from the channel on the right reaching the confluence junction, leaving an air segment (airlock). (*e*) An illustration diagram of each part of the airlock microfluidic design

The design and layout of the Gene-Z prototype was similar to that described previously (Stedtfeld et al. 2012), with minor modifications including: i) embedding optical fibers in an apparatus placed above the card (previously, fibers were embedded below the card), ii) cards were inserted into the Gene-Z device via a port on the side of the device (instead of a door at the top, Fig. 1). Advantages of this arrangement included: i) elimination of occasional misalignment of cards with the optics, ii) reduction in cost of manufacturing the aluminium heater (i.e. drilling negative features for alignment of the shelled card and angled holes for optical fibers in the aluminium heat sink), and iii) elimination of pressure requirements needed by the door to obtain equal contact between thin non-rigid cards and the heater.

After loading the card with sample, and securing the screw cap, cards were inserted into Gene-Z, and the device was started using an iPod Touch as described previously (Stedtfeld et al. 2012). All reactions were run for 60 to 90 min and data was sent from the iPod to the user’s email account for further analysis. Data analysis was performed as previously described to calculate the threshold time (Tt), akin to threshold cycle. The signal to noise ratio (SNR) was calculated as the signal at a given time point minus the median signal at the start of the reaction, divided by the standard deviation of the signal at the start of the reaction. Results testing replication of the device optics and heater temperature along the length of the heater of the Gene-Z device have been reported previously (Stedtfeld et al. 2012).

### Endpoint images of LAMP reactions in microfluidic card

Endpoint images of the LAMP reaction within the microfluidic card were captured using either a cell phone camera (Galaxy Note II, Samsung), or a digital camera (Cybershot, Sony). SYTO intercalating dye was excited using a 530 nm green LED (05027-PM12, LED Supply). A 572 ± 20 nm bandpass filter (FF01-572/28-25, Semrock) was fixed to capture emission signals from wells. For experiments with 40 % blood, a 0.25 megapixel monochrome CCD camera (MEADE DSI Pro, Irvine, CA, 1.0 s exposure time) was necessary to capture increased signal from an amplification event.

## Results and discussion

A card, termed “airlock” was developed that does not require peripheral systems, permits straightforward operation via loading with a syringe or micropippette, and can be used with either low cost photodetectors, CCD cameras, or the Gene-Z device for rapid and real-time optical readout. Cards were tested for proper loading using blood, urine, and saliva spiked with isolates from septic infections. LAMP was performed in the cards to ensure predispensed primers were not carried to subsequent wells during sample filling, and no observable diffusion between wells during the reaction. Experiments also tested analytical sensitivity and replication of the non-functionalized native acrylic cards.

### Principle of sample distribution in the card

The airlock mechanism (Fig. 1b, supplemental video 1) works as follows: (a) sample bifurcates into left and right channels. (b) Prior to sample filling the channel to the right of the junction, the large volume well on the left fills due to the hydrophobic surface of native acrylic. (c) As the well fills with sample, liquid on the right channel nearly reaches a confluence junction. (d) The liquid completely fills the reaction well and only a portion of the channel downstream of the well, which becomes blocked due to liquid in the right of the confluence junction. A plug of air termed airlock is formed between the channel downstream from the well and the channel on the right side of the confluence junction. (e) This permits distribution of sample into a series of wells without downstream carryover of primers. The channel succeeding the well has a longer flow path and smaller dimensions to further prevent downstream carryover from the well.

The hydrophobic surface of native acrylic (contact angle of 75 to 80°, Raghavan et al. 2000; Tsao et al. 2007), encourages loading of sample into larger reaction wells preceding flow into the smaller dimension channel to the right of the confluence junction. The utility of the native acrylic surface was further tested by inducing a hydrophillic layer, as described by Tsao and coauthors (Tsao et al. 2007) within the channels of the card. Sample preferentially flowed into channels to the right of the junction in the hydrophillic card, causing incomplete filling of wells, which behaved as capillary valves (Glière and Delattre 2006). To our knowledge, the card is unlike a majority of microfluidic models described for POC diagnostics, in which capillary forces are utilized (Rafati and Gill 2015) and induced (Gervais and Delamarche 2009) for passive flow. The utility of surface tension to manipulate fluid flow, and allow for simplified loading (that only requires a micropipette) has been described previously via immersion of water and oil for nucleic acid sample purification (Berry et al. 2011) and proteomics diagnostics (Casavant et al. 2014). However, the airlock card expands the utilization of surface tension via bifurcated channels and the airlock mechanism for sample displacement into multiple wells.

**Fig. 2:**
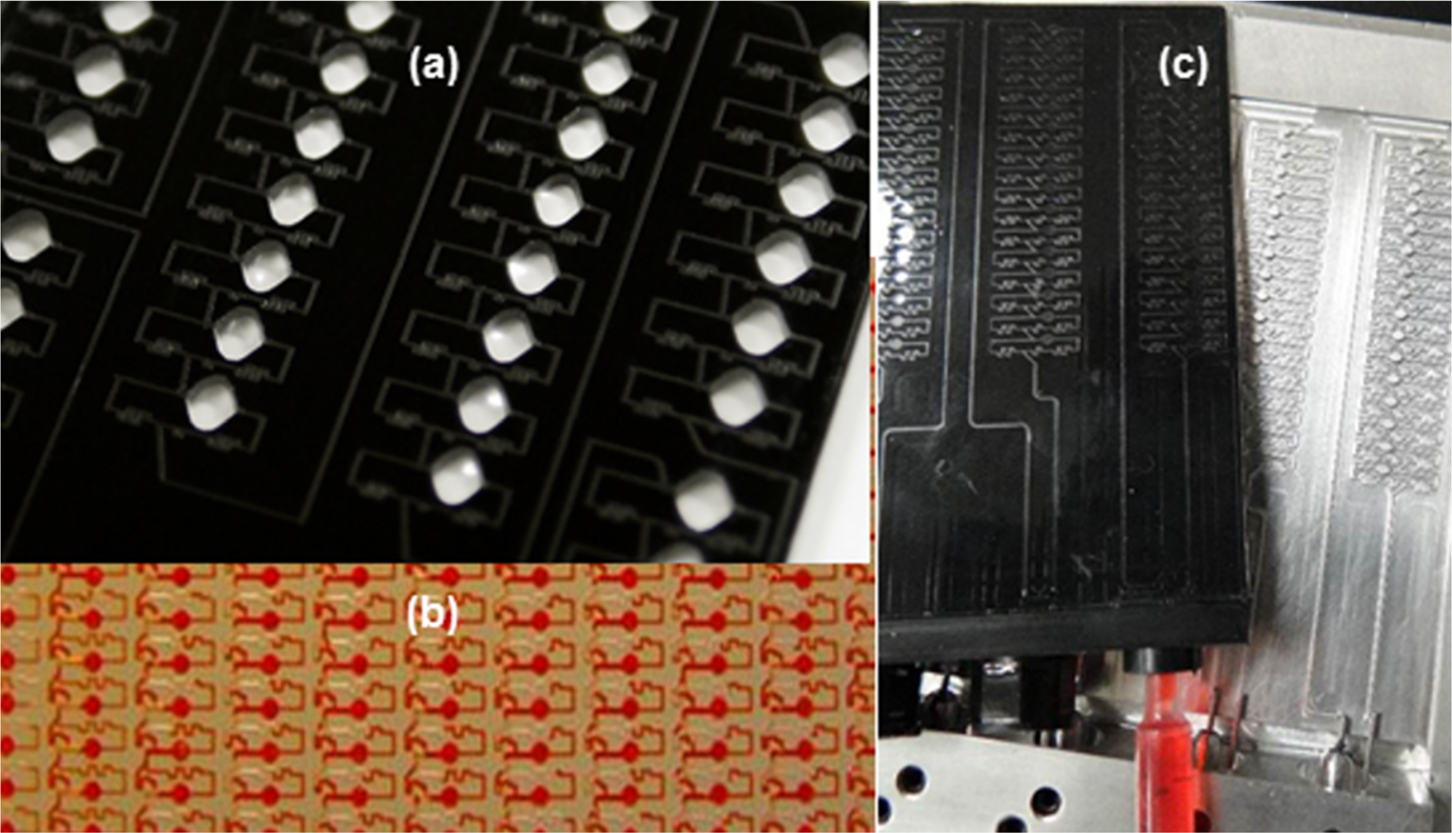
Cards with different level of throughput and fabrication techniques. **a** A 32 reaction well card with 25 μL volumes etched with the hobby laser. **b** A portion of a card with 1536 reaction wells etched with the CO_2_ laser. **c** A mold used to test fabrication of 64 well cards via injection molding

The single-etched layer of the microfluidic card also reduces costs and complexity of microfabrication. All channels were etched with greater than or equal to 200 μm dimensions to prevent clogging with dust and permit fabrication outside of a clean room. Total material costs of the acrylic layer and silicon adhesive tape is approximately $0.83/card. Costs required for reaction constitutes is approximately $0.043/ μL, which is $6.93 to fill the 64 reaction well card. For producing the airlock card on a more cost effective basis (without the CO_2_ laser), the 64 well version of the airlock card was also tested for fabrication via injection molding (Benchtop Injection Molding Machine, Medium Machinery LLC) with polypropylene pellets (PPBLK, LNS Technologies) casted directly with loading ports that can be sealed with screw-caps (mold fabricated by Herman Engineering, Fig. 2c). Mass production replicate techniques such as embossing and injection molding have been described previously for reducing microfluidic fabrication costs (Dang et al. 2005; Martynova et al. 1997). Tests with both acrylic and polypropylene cards fabricated with lasers and injection molding further demonstrate that the airlock card may be adaptable to numerous plastic substrates. For even lower detection costs, endpoint images can be captured (for presence/absence) using a CCD camera, which only requires a green LED and orange filter lenses for excitation and emissions, respectively.

The laser is also well suited for rapid development of microfluidic cards with design iterations competed within 5–10 min. Such tools are expected to complement the next generation of biosensors (Chen et al. 2008). Flexibility of the desktop CO_2_ hobby laser also permits fabrication of cards with varying throughput including 8, 32, 64, 384 and 1536 wells with reaction well volumes ranging from 0.5 to 25 μL (Fig. 2a and b).

### Analyte cross-reactivity and diffusion

**Fig. 3:**
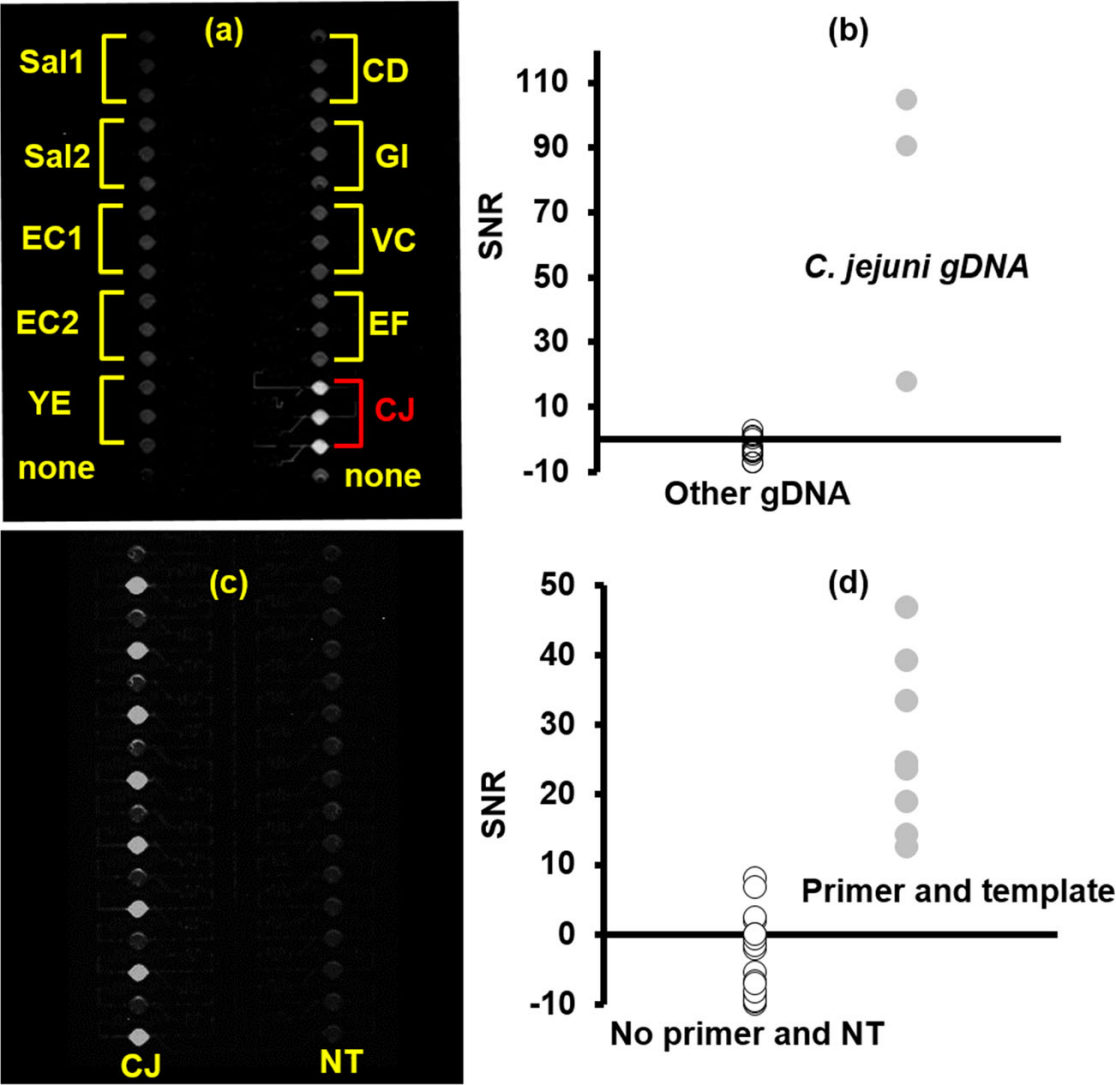
Testing cards for primer and DNA cross-reactivity during filling or diffusion. **a** A CCD image of the card captured after the 60 min LAMP reaction. Two lanes were predispensd with dehydrated gDNA from multiple organisms (1.4 ng of gDNA from each organism, equivalent to 812,000 genomic copies/ reaction for *C. jejuni)*. Abbreviations for organism from other predispensed templates are listed in material and methods. Once assembled, lanes were loaded with LAMP containing primers associated with *C. jejuni*. **b** SNR of all 32 wells as measured with the Gene-Z device, *black open dots* are wells without targeted gDNA, and *gray dots* are the three wells predispensed with gDNA from *C. jejuni* (CJ). **c** Two lanes predispensed with primers targeting *C. jejuni* in every other well. Once assembled, one lane was loaded with LAMP containing *C. jejuni* gDNA (812,000 genomic copies/reaction), and the other lane was loaded with LAMP mixture containing a no-template control (NT). **d** SNR of chip predispensed with primers as measured with the Gene-Z device, *black open dots* are wells that were not predispensed primers and the no template control lane, and *gray dots* are the eight wells predispensed with primers and loaded with LAMP reaction containing *C. jejuni* gDNA

The airlock card was tested for 1) primer carryover during sample loading, 2) diffusion of primers, amplicons, and gDNA during LAMP, and 3) interference of fluorescent emissions between adjacent wells. In one card, gDNA from multiple target organisms was pre-dispensed and dehydraed in two of the lanes (Fig. 3a), and subsequently loaded with a no-template LAMP reaction mixture that contained primers targeting the mapA gene in *C. jejuni*. The other two lanes were predispensed with primers targeting the mapA gene in adjacent wells (Fig. 3c); and one lane was loaded with gDNA from *C. jejuni*, and the second with a no template reaction. The measured SNR was less than 10 in all wells not predispensed with target gDNA or primers (Fig. 3b and c) and greater than 10 in targeted wells. This indicates no measurable movement of primers and gDNA between wells due to diffusion or sample loading, and no measurable interference of fluorescent emission between adjacent wells. As described previously (Fang et al. 2011; Ramalingam et al. 2010; Stedtfeld et al. 2012), diffusion is limited due to constraints of channel dimensions and reaction time.

**Fig. 4:**
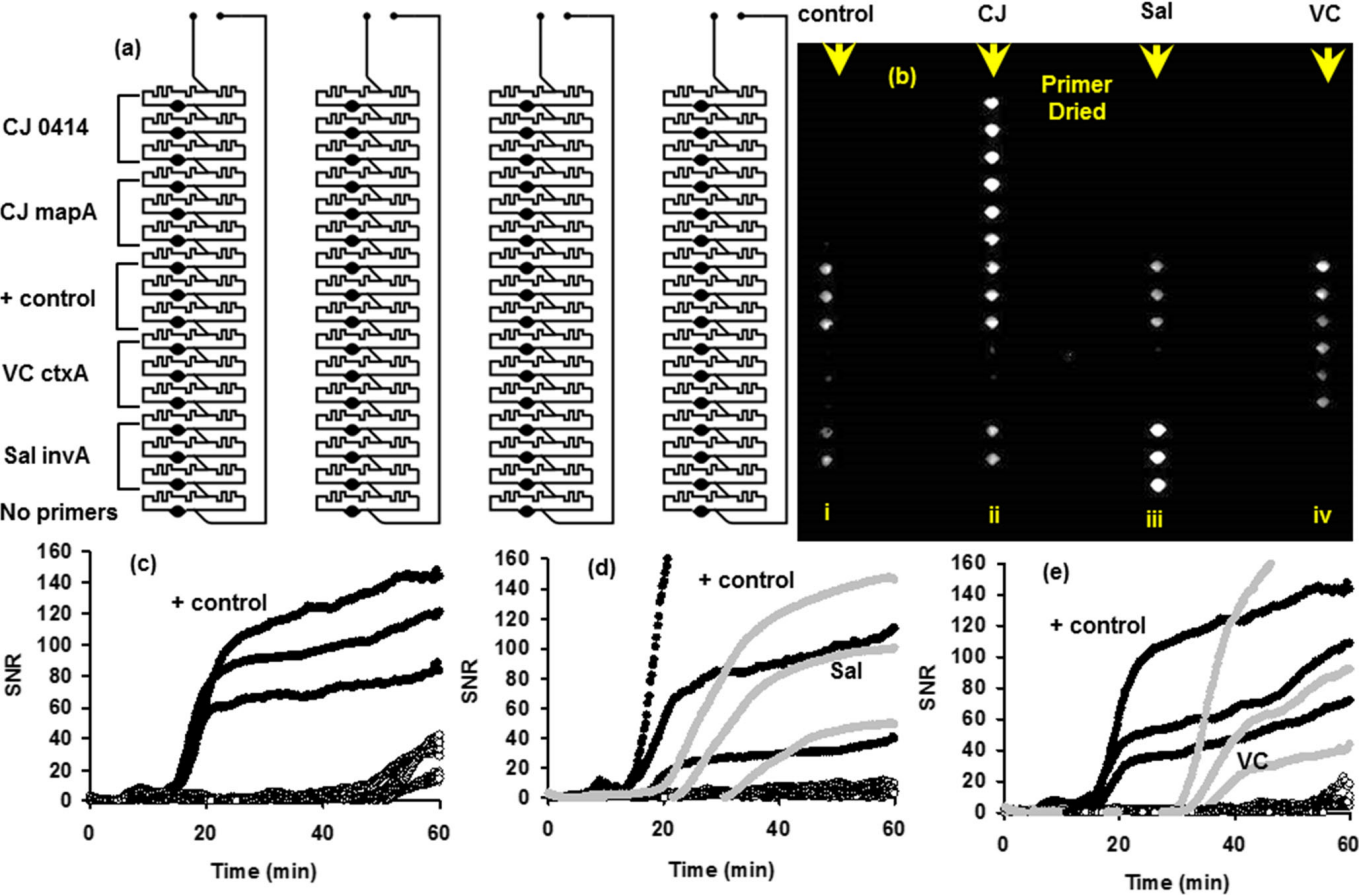
Testing cards for LAMP based detection of multiple targets in parallel. All lanes were spiked with an internal positive control (gDNA from a plant pathogen) spiked into all samples plus templates of gDNA from CJ (*C. jejuni*, 34,800 genomic copies/reaction) was added to lane 2 (ii), Sal (*Salmonella* 11,100 genomic copes/ reaction) into lane 3 (iii), and VC (*V. cholera*, 13,800 genomic copies/reaction) into lane 4 (iv). **a** Map showing how primers were dried into wells, and **b** picture of card (captured with exposure control CCD) after the 60 min LAMP reaction. **c**, **d**, **e** SNR of all wells in lanes i(**c**), iii(**d**), iv(**e**) versus reaction time as monitored with the Gene-Z device

Cards were also tested for simultaneous amplification of multiple targets. Primers targeting four separate functional genes from *V. cholerae*, *C. jejuni*, and *Salmonella* were each dispensed and dehydrated in triplicate. The same configuration of dried primers (Fig. 4a) was placed into each of four lanes on a 4-sample card, and each lane was loaded with gDNA with an equivalence of 34,800 genomic copies/reaction for *C. jejuni*, 13,800 genomic copies/reaction of *V. cholerae,* and 11,100 genomic copes/reaction of *Salmonella*. Genomic DNA from a plant pathogen was spiked as an internal positive control into all samples for a final concentration of 5.7 pg/μL (~60 copies/reaction well). The card was monitored in real time on the Gene-Z device. Following the 60 min incubation time, an image was captured using the high exposure CCD (Ahmad et al. 2011). Amplification curves from three of the lanes show signal measured with the Gene-Z device during the reaction (Fig. 4c, d, and e).

An increase in signal was only observed in wells with targeted primers and not in adjacent non-targeted wells. Wells with primers targeting *Salmonella* showed an average SNR of 14.1 when not targeted, however the average SNR was 99.3 when targeted. The slight increase in signal when not targeted was thought to be the result of primer-dimers as the average time to positive amplification was 53 min. When gDNA for *Salmonella* was present in the sample, an average Tt of 26 min was observed. This requires redesign of the *Salmonella* primers and is not related to sample dispensing aspects of the card.

It should be noted that the airlock card is only optimized for isothermal amplification. When the card was cycled (with temperatures typically used for qPCR), gas inside the airlock segment expands and contracts with heating and cooling, which often moves large bubbles into reaction wells. Perhaps the movement of air under thermal cycling conditions could be accommodated in a modified design.

### Analytical sensitivity and reproducibility

The high surface to volume ratio of native acrylic was also evaluated for LAMP of low abundance targets. For this experiment, a LAMP primer targeting the stx2 gene from *E. coli* was loaded and dried in every other well of two separate cards, each fabricated so that four samples could be loaded on each card (16 wells per sample, 8 wells per sample predispensed with primers). Seven of the eight lanes were loaded with a reaction mixture spiked with dilutions of gDNA from *E. coli*, and a no template control was added to the eighth lane. Reaction mixtures with the same mass of gDNA per sample were also run in three replicate vials in a conventional real-time thermocycler (Chromo4^™^, BioRad) under isothermal conditions.

**Fig. 5:**
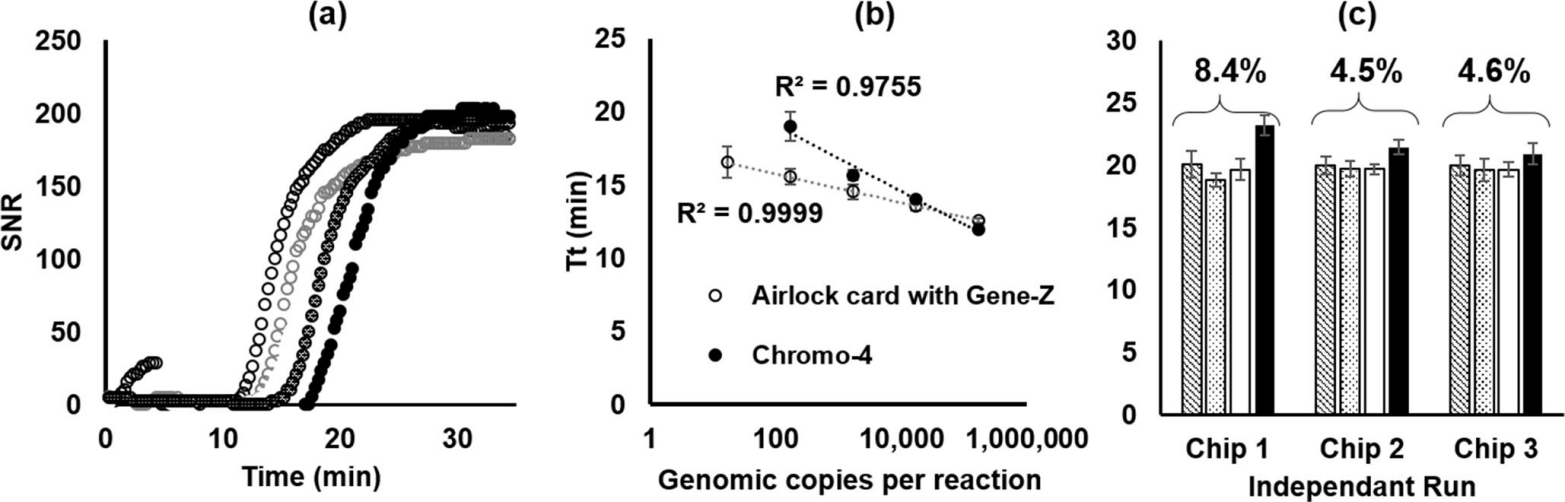
Analytical sensitivity and reproducibility of airlock cards tested with LAMP reactions. **a** Real time LAMP profiles captured with the Gene-Z from one well from each lane of the microfluidic card loaded with serial dilutions of template gDNA. **b** Average time to threshold (Tt) for replicates that amplified for reactions tested with isothermal temperatures in a conventional thermocycler (*n* = 3) and the airlock card (*n* = 8). *Error bars* represent standard error. **c** Mean, standard deviation, and coefficient of variation (in *parenthesis*) of the T_t_ for all reaction wells in each of the four lanes tested in three separate cards

Results showed comparable linearity for dilutions tested with the card and in vials on a conventional thermocycler (Fig. 5). Lower dilutions of gDNA tested on the microfluidic card (monitored with the Gene-Z) had a slightly shorter Tt than reactions tested on the conventional thermocylcer. A detection limit of approximately 20 genomic copies/reaction well was observed for the *E. coli* stx2 assay on the card. However, only 3 out of 8 wells showed amplification with 20 genomic copies/reaction. Tests in the Chromo4^™^ thermocycler resulted in positive amplification in 0 out of 3 vials at 20 gDNA copies/reaction.

While the stx2 assay lacked single copy detection limit, the microfluidic card provides a level of sensitivity that is comparable to reactions tested in vials with the thermocycler. We routinely observe that amplification efficiency of LAMP in native polymer cards (COP and polyester) is similar to that in tubes (Ahmad et al. 2011; Stedtfeld et al. 2012). The card is expected to have a sensitivity dependent upon the assay, which has been previously shown to allow quantification of single digit copy numbers/reaction (Thekisoe et al. 2010; Tsai et al. 2009; Yamazaki et al. 2008).

Inter and intra assay reproducibility were also tested to ensure that the thicker ~1.59 mm card did not influence temperature distribution along the length of the card and ultimately influence threshold time and quantification. LAMP primers were predispensed into all reaction wells on three separate microfluidic cards and loaded with approximately 5 × 10^6^ genomic copies/reaction. Results show a coefficient of variation of 6.1 % between all three cards (Fig. 5c), which is comparable to thinner cards that were previously described with a coefficient of variation of 7.8 % (Stedtfeld et al. 2012).

### Testing cards with blood, urine, saliva

The airlock card was tested for correct loading of body samples (blood, urine, and saliva) spiked with dilutions of bacterial isolates derived from septic infections. Ultimately, decentralized genetic testing must also allow for sample-in-results-out, with automation, minimization, or elimination of sample preparation (DNA extraction/purification). LAMP and direct amplification have been previously demonstrated with many different targets (bacterial, viral, human, fungal), and sample types with varied concentrations in the amplification reaction including 1 to 50 % blood (Curtis et al. 2009; Ebbinghaus et al. 2012; Masaomi et al. 2003; Patterson et al. 2013; Soejima et al. 2011), 10 % stool, (Francois et al. 2011), 20 % urine samples (Hill et al. 2008; Koizumi et al. 2012), and 32 % nasal swabs (Nie et al. 2012). As such, the airlock cards were tested with these sample types at varying sample concentrations in the final LAMP reaction mixture.

Blood sample experiments consisted of spiking blood into 1, 5, 10, and 40 % of the total amplification reaction volume and loading cards. In total, 635 out of 640 wells loaded properly with the tested blood samples (Table 1, Fig S1–S3). It should be noted that wells that did not load properly seemed to be due to inadequate cleaning of the card, with sample not filling wells completely. The 40 % blood sample loaded slower than all other samples, requiring 2.5 min to completely fill a 16 well lane. Six cards were tested with urine samples spiked with dilutions of GBS isolates at 10, 25, and 40 % concentration of the final LAMP reaction mixture, and 382 out of 384 wells loaded properly (Fig S4–S5). Five cards were tested with saliva samples spiked with dilutions of GAS isolates were tested as 10 and 25 % concentrations of the LAMP reaction mixture, and 315 out of 320 wells loaded properly (Fig S6).

### Sensitivity and selectivity of bacteria spiked into body matrices

Assays designed to target lmb, cfb, and scpA genes in GBS and GAS amplified down to single digit CFU/reaction when spiked into urine and saliva (tested as 10 and 25 % of the reaction, Table 1). Considering a 4 μL reaction well, assuming 25 % of the LAMP reaction is sample, and a detection limit of approximately 1 CFU/reaction; the limit of detection is approximately 1000 CFU/mL. This level of sensitivity is below the criterion commonly used for defining significant bacteriuria, which is 10^5^ CFU/mL in urine (Stamm et al. 1982; Rosa et al. 2010), and 10^4^–10^7^ CFU/mL for conventional bacterial loads of GAS obtained via throat swabs (Dunne et al. 2013). Thus, diagnostics of urine and throat swabs could be performed with minimal sample preparation, potentially allowing genetic diagnostics in a decentralized setting. While demonstrated directly with saliva, elution from swabs is expected to have a lesser influence on inhibition.

Assays designed to target nuc and uidA amplified down to approximatly 10–100 CFU/reaction with 5 % blood (Table 1), corresponding to a detection limit of 10^4^ to 10^5^ CFU/mL. For clinically relevant blood samples with lower bacterial concentrations (e.g., 1–100 CFU/ml in adults (Yagupsky and Nolte 1990) and <10 CFU/ml in neonates (Reier-Nilsen et al. 2009)), this approach still poses a challenge due to the volumetric constraints of the cards. However, a concentration step could be used to capture bacteria (Mai et al. 2013; Cooper et al. 2014), or a commercially available kit such as the MolYsis or Looxster kits for concentrating cells in blood (Wiesinger-Mayr et al. 2011; McCann and Jordan 2014). Another potential option is to centrifuge at a low speed (500*g* for 5 min) to remove blood cells, followed by a high speed (5000*g* for 10 min) to concentrate bacteria from plasma (Larsson and Bergstrom 2008). While more hands-on-time may be required for these concentration steps, the card may still serve as downstream tool for parallel diagnostics.

Presence and absence of LAMP assays agreed with conventional culture based methods in terms of identifying isolates. Overall, 23 LAMP assays were tested with 11 isolates from septic patients. Results of testing each assay and isolate showed 99.6 % specificity with culture based identification (Table S3). Specificity of LAMP in cards was also comparable to presence and absence results observed using conventional vials in the Chromo4^™^ (Table 1, S3). For example, only the uidA, coa, and nuc assays amplified in the blood samples spiked with *E. coli* and *S. aureus* isolates, which was in accordance with LAMP reactions on the Chromo4^™^ (Fig S2, S3). For the 10 and 25 % concentration of urine and saliva, assays targeting the lmb, mstA, cfb, and scpA amplified in accordance with the GBS and GAS isolates tested on the Chromo4^™^ (Fig S5). However, reactions tested with urine as 40 % of the LAMP reaction mixture did not amplify, which we suspect is due to sample inhibition caused by higher salt content.

### Testing card with laboratory prepared mock fluids

Cards were also tested with laboratory prepared mock samples containing water and varying concentrations of glycerol for viscosities of 0.69, 0.89, 1.01, 1.40, and 1.80 mPa•s and red dye for visualization. The concentration of glycerol used for various viscosities has been described previously (Cheng et al. 2008). Viscosities were chosen to mimic human serum and blood plasma, which varies from 1.27 to 1.75 mPa•s for 21 to 37 °C (Haidekker et al. 2002; Rosencranz and Bogen 2006; Rosenson et al. 1996). Each solution was loaded in a card containing a single inlet to all 64 wells. A CCD was used to record sample flow during loading, and to acquire an end-point image after loading. The airlock formed uniformly with all 64 wells filled with varying sample viscosities (Fig S7). Time required to load the entire card of 64 wells increased slightly with the increase in viscosity which was 37, 40 39, 50, and 60 s with viscosities of 0.69, 0.89, 1.01, 1.40, and 1.80 mPa•s, respectively.

The influence of sample surface tension was also tested. Three sample mixtures of varying concentrations of Tween 20 in water and red dye were prepare to have a surface tension of 50, 60, and 70 N/m (Nino and Patino 1998; Patist et al. 2000). These surface tensions were chosen to mimic human serum and plasma, which varies from 52 mN/m at 37 °C to 59–60 mN/m at 20 °C (Clark 1928; Rosina et al. 2007). Solutions were loaded into separate 64-well airlock cards (with one inlet port) and images were taken using a CCD camera after loading and during filling (Fig S8). All tested surface tension resulted in uniform airlock formation in all 64 wells. Time to load the cards did not change significantly, requiring 37, 36, and 38 s for 50, 60, and 70 mN/m surface tension, respectively.

In total, 28 cards were tested with blood, urine, saliva, and laboratory prepared mock samples. Within the tests performed here, the airlock mechanism was stable and wells loaded correctly in 99.3 % of instances. Overall, tests demonstrate that the airlock card can be used with various sample types, manufacturing techniques, and diagnostics applications requiring multiple analytes.

## Electronic supplementary material

supplemental video 1 Video showing close-up of airlock mechanism for simplified sample dispensing into multiple wells. (MP4 489 kb)

Table S1 (DOCX 19 kb)

Table S2 (DOCX 15 kb)

Table S3 (DOCX 19 kb)

Fig S1 (DOCX 412 kb)

Fig S2 (DOCX 534 kb)

Fig S3 (DOCX 836 kb)

Fig S4 (DOCX 347 kb)

Fig S5 (DOCX 462 kb)

Fig S6 (DOCX 396 kb)

Fig S7 (DOCX 19708 kb)

Fig S8 (DOCX 11200 kb)
