# Supplementary material for: Static self-directed sample dispensing into a series of reaction wells on a microfluidic card for parallel genetic detection of microbial pathogens
Source: Biomed Microdevices. 2015 Aug 11;17(5):89. doi: 10.1007/s10544-015-9994-1 (PMC4531140; doi:10.1007/s10544-015-9994-1)
Supplement: Supplementary file 2 — (DOCX 19 kb) [file 10544_2015_9994_MOESM2_ESM.docx]

**Table S1.** Sequences of primers used to test the cards and direct amplification.

| **Pathogen/gene** | **Primer** | **Sequence (5'-3')** |
| --- | --- | --- |
| ***C. Jejuni*** | | |
| *Cj0414a* | F3 | GCAAGACAATATTATTGATCGC |
|  | B3 | CTTTCACAGGCTGCACTT |
|  | FIP | ACAGCACCGCCACCTATAGTAGAAGCTTTTTTAAACTAGGGC |
|  | BIP | AGGCAGCAGAACTTACGCATTGAGTTTGAAAAAACATTCTACCTCT |
|  | LF | CTAGCTGCTACTACAGAACCAC |
|  | LB | CATCAAGCTTCACAAGGAAA |
| *mapA* | F3 | ACAAGATACTTTTGCTCAAGTT |
|  | B3 | GCATTAAAATTCACATCRACAA |
|  | FIP | AACATCGCTAATGTATAAAAGCCCTCTCAATGCAGTTCTTGTGAAAG |
|  | BIP | TTCAATGTTGTGCCAATAAACGCTTTAAAACCTTTTGCYCTTCT |
|  | LF | TTTGCTTCAAAACCACCAGGA |
|  | LB | CTGGTATTGCTTTGAAAAAGGTTTATTTAC |
| ***Salmonella*** | | |
| *invA* | F3 | CGGCCCGATTTTCTCTGG |
|  | B3 | CGGCAATAGCGTCACCTT |
|  | FIP | GCGCAGCATCCGCATCAATAATATGGTATGCCCGGTAAACAG |
|  | BIP | GAACGGCGAAGCGTACTGGACATCGCACCGTCAAAGGAA |
|  | LF | CCTTCAAATCGGCATCRATACTCAT |
|  | LB | AAGGGAAAGCCAGCTTTACG |
| ***V. cholerae*** |  |  |
| *ctxA* | F3 | CCCCACCTTTAACTAGAACAA |
|  | B3 | GCCAAATCCTTTGCTATCGA |
|  | FIP | CGCAGGGCGATTTTCAAAAATTAAACAATAATGCAGYAAATGGGAT |
|  | BIP | TTAACCATTTTAGGCCCTAGCGGCAAATCCAATTTCCTTGTGCT |
|  | LF | GCTTCGTCTTTAAAGYGAGGATTG |
|  | LB | AGCAGCTTTAACGGTTTGGG |
| *toxR* | F3 | CGAGTGGAAACGGTTGAAGA |
|  | B3 | AGGGGAAGTAAGACCGCTAT |
|  | FIP | GCACACTGCTTGAYTCTGCGTACGAAAGCGAAGCTGCTCAT |
|  | BIP | AGCCACTGTAGTGAACACACCGTCGATTCCCCAAGTTTGGAG |
|  | LF | ACAGATTCTGGCTGAGAGATGTC |
|  | LB | CAGCCAGCCAATGTTGTGAC |
| ***E. coli*** | | |
| stx2 | F3 | GAGATATCGACCCCTCTTG |
|  | B3 | AATCTGAAAAACGGTAGAAAGT |
|  | FIP | TCCACAGCAAAATAACTGCCCAACATATATCTCAGGGGACCA |
|  | BIP | GATGTCTATCAGGCGCGTTTTGCCGTATTAACGAACCCGG |
|  | LF | TGTGGTTAATAACAGACACCGATG |
|  | LB | ACCATCTTCGTCTGATTATTGAGC |
| uidA | F3 | TATCTACCGCTCGCGTCG |
|  | B3 | CGAGCATCTCTTCAGCGT |
|  | FIP | TCCTTTGCCCGAATCGCATCTTAGTGAAGGCGAACAGTTCC |
|  | BIP | TCGATAACGTGCTGATGGTGCATGCGAGTCGGTAGGGTTG |
|  | LF | CGTAAAGTAGAACGGTTTGTGGTTA |
|  | LB | CACGCATAATGGACTGGATTGG |
| ***S. aureus*** | | |
| coa | F3 | GATGCTGGTACAGGTATYC |
|  | B3 | TTTGCATGTGTTGTTACGT |
|  | FIP | GCRTTTGTTTCTGATGGCTTATTGAGTGAATACAACGATGGAACAT |
|  | BIP | TAACGACAAATCAAGATGGCACAGCATTTGTTTTGCTTGGTTTG |
|  | LF | TCTTGGTCTCGCTTCATATCCAA |
|  | LB | GTAWCATATGGCGCTCGCCCAA |
| nuc | F3 | AACAGTATATAGTGCAACTTCAA |
|  | B3 | CTTTGTCAAACTCGACTTCAA |
|  | FIP | ATGTCATTGGTTGACCTTTGTACATAAATTACATAAAGAACCTGCGA |
|  | BIP | TATTGGTKGATACACCTGAAACAAAATTTTTTTCGTAAATGCACTTGC |
|  | LF | ATTTAACCGTATCACCATCAATCGC |
|  | LB | AGGTGTAGAGAAATATGGTCCTGAA |
| mecA | F3 | ATCTCATATGCTGTTCCTGTA |
|  | B3 | AAAAAACGAGTAGATGCTCAA |
|  | FIP | AATGCAGAAAGACCAAAGCATACATGCCAATTCCACATTGTTTCG |
|  | BIP | TGACGCTATGATCCCAATCTAACTACTACGGTAACATTGATCGC |
|  | LF | TTTAAAATCAGAACGTGGTAAAATTTTAGAC |
|  | LB | CCACATACCATCTTCTTTAACAAAATTAAATTG |
| ***Streptococcus*** | | |
| cfb | F3 | TGTATAGATTGTAGCTCTATCAGTT |
|  | B3 | AAGCCTTAACAGATGTGATTG |
|  | FIP | TCCATTTGCTTCAGTTGATTCAATTCAGGATAAGTTAAAACCTTTTGTTC |
|  | BIP | TGCGAATAACCAGCTTAGTTATCCCACTTTTTCAACTCAACATTTAGC |
|  | LF | GCTCAAGTTAACGATGTAAAGGCATTA |
|  | LB | TCCCATATCAATATTTGCTTGACTAACC |
| mstA | F3 | GCTGATGTGATTTTCTATAATGGTA |
|  | B3 | CAATCAATTGTTTGGCAATGT |
|  | FIP | ACGGCAAAGTAATCTTTGTTTTTCGCAATCTAGAAGATGGTGGGC |
|  | BIP | ACTTGGAAGGTGCAAGCGAAAAATGATTCCGTTTTCGAGAT |
|  | LF | GCATTTTTCACTAGTTTGGTGAACC |
|  | LB | GAAAAGAAGATCCACATGCTTGGT |
| scpA | F3 | CACACGTGTCAGGGATCT |
|  | B3 | CCTTAGCTCCCAAGTTGAC |
|  | FIP | TCAGGCATCGCACCTTCTAGTGTCAGGAAATGCTCCAT |
|  | BIP | TCAATTGCTTTTGATGCGTGTCTCTCTGATAGCTTGAGCGTA |
|  | LF | GCGGTAAGGTTCTTTCGTTTCAG |
|  | LB | AATGGACTAGCAGACTATGCTCGTA |
| lmb | F3 | CACAAGGCATTGACCCTG |
|  | B3 | GCACCTTTTTAAATTTTTGAGTG |
|  | FIP | AGCTCTTTAGCGATATTAACAGCTTTTTATGACCCACATACCTGG |
|  | BIP | AGGACGTTTGGATCCTAAACACAATCTTCAGTTAGTTGCTCTGC |
|  | LF | CCAGCTAAAACGGGATCCGT |
|  | LB | ACAGTTACACTAAAAAGGCTAAGGC |
