# Supplementary material for: Static self-directed sample dispensing into a series of reaction wells on a microfluidic card for parallel genetic detection of microbial pathogens
Source: Biomed Microdevices. 2015 Aug 11;17(5):89. doi: 10.1007/s10544-015-9994-1 (PMC4531140; doi:10.1007/s10544-015-9994-1)
Supplement: Supplementary file 3 — (DOCX 15 kb) [file 10544_2015_9994_MOESM3_ESM.docx]

**Table S2.** Initial test to verify specificity of LAMP assays and identity of bacterial isolates. Threshold time (T_t_) for LAMP assays with gDNA extracted from clinical isolates tested in conventional vials (Chromo4^TM^ Biorad) under isothermal condition. Type strains(*) were spiked to have a total mass of 1 ng per reaction, and 2 μl was added from isolate lysates yielding 472, 24, 22, 53 ng per reaction for *S. aureus*, GAS, GBS, *E. coli*, respectively. Numbers indicate average time to positive amplification (min) from duplicate reactions.

| Isolate/Assay | lmb | mstA | cfb | scpA | stx2 | uidA | coa | mecA | nuc |
| --- | --- | --- | --- | --- | --- | --- | --- | --- | --- |
| *S. aureus* | - | - | - | - | - | - | 23.98 | - | 14.5 |
| *E. coli* | - | - | - | - | - | 20.6 | - | - | - |
| GAS | 16.9 | 27.7 | - | 17.5 | - | - | - | - | - |
| GBS | 13.6 | - | 16.8 | 25.7 | - | - | - | - | - |
| **S. agalactiae* | 10.8 | 32.8 | 16.2 | 21.8 | nt | nt | nt | nt | - |
| **E. coli* O1:57:H7 | nt | nt | Nt | nt | 14.9 | 13.6 | nt | nt | - |
| **S. aureus* Mu50 | nt | nt | Nt | nt | nt | Nt | 32.2 | 15.5 | 13.5 |

nt: not tested
