# Supplementary material for: Static self-directed sample dispensing into a series of reaction wells on a microfluidic card for parallel genetic detection of microbial pathogens
Source: Biomed Microdevices. 2015 Aug 11;17(5):89. doi: 10.1007/s10544-015-9994-1 (PMC4531140; doi:10.1007/s10544-015-9994-1)
Supplement: Supplementary file 4 — (DOCX 19 kb) [file 10544_2015_9994_MOESM4_ESM.docx]

**Table S3.** Testing specificity of 23 LAMP assays against gDNA of 11 bacterial isolates from septic infections. Calls are based on 2 of 2 replicates showing amplification in vials tested using the Chromo-4^TM^. Cells with “+” and “-“ indicate true positive and true negative, respectively. Cells with “u” indicate presence or absence of the target gene is unknown for the isolate tested, and the gray cell indicates false positive.

|  | *Corynebacterium spp.* | *S. aureus* | MRSA | Coag. negative *Staphylococcus* | Group A Streptococcus | Group B Streptococcus | VSG (Viridans-Streptococcus) | *P. mirabilis* | *E. coli* | *K. pneumoniae* | *Enterococcus* |
| --- | --- | --- | --- | --- | --- | --- | --- | --- | --- | --- | --- |
| **uidA** | - | - | - | - | - | - | - | - | + | - | - |
| **stx1** | - | - | - | - | - | - | - | - | -u | - | - |
| **stx2** | - | - | - | - | - | - | - | - | -u | - | - |
| **eaeA** | - | - | - | - | - | - | - | - | -u | - | - |
| **bacA** | - | - | - | - | - | - | - | - | - | - | - |
| **lmb** | - | - | - | - | + | + | - | - | - | - | - |
| **cfb** | - | - | - | - | - | + | - | - | - | - | - |
| **scpA** | - | - | - | - | + | + | - | - | - | - | - |
| **ssa** | - | - | - | - | + | - | - | - | - | - | - |
| **mstA** | - | - | - | - | + | - | - | - | - | - | - |
| **ef0027** | - | - | - | - | - | - | - | - | - | - | + |
| **ace** | - | - | - | - | - | - | - | - | - | - | + |
| **sodA** | - | - | - | - | - | - | - | - | - | - | - |
| **vicK** | - | + | + | - | - | - | - | - | - | + | - |
| **nuc** | - | + | + | - | - | - | - | - | - | - | - |
| **coA** | - | + | + | - | - | - | - | - | - | - | - |
| **mecA** | - | - | + | +u | - | - | - | - | - | - | - |
| **uge** | - | - | - | - | - | - | - | - | - | + | - |
| **wabG** | - | - | - | - | - | - | - | - | - | + | - |
| **dtxR** | -u | - | - | - | - | - | - | - | - | - | - |
| **exoS** | - | - | - | - | - | - | - | - | - | - | - |
| **popD** | - | - | - | - | - | - | - | - | - | - | - |
| **oprL** | - | - | - | - | - | - | - | - | - | - | - |
