# Supplementary material for: Static self-directed sample dispensing into a series of reaction wells on a microfluidic card for parallel genetic detection of microbial pathogens
Source: Biomed Microdevices. 2015 Aug 11;17(5):89. doi: 10.1007/s10544-015-9994-1 (PMC4531140; doi:10.1007/s10544-015-9994-1)
Supplement: Supplementary file 5 — (DOCX 412 kb) [file 10544_2015_9994_MOESM5_ESM.docx]

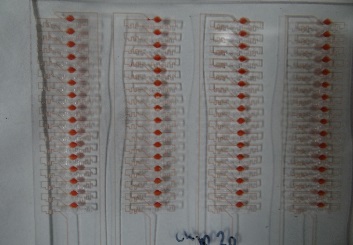

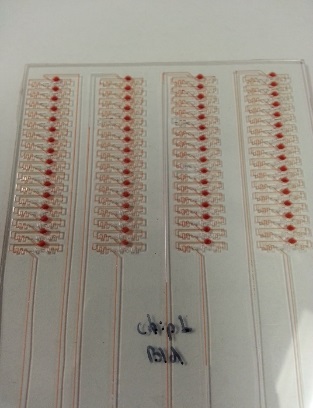

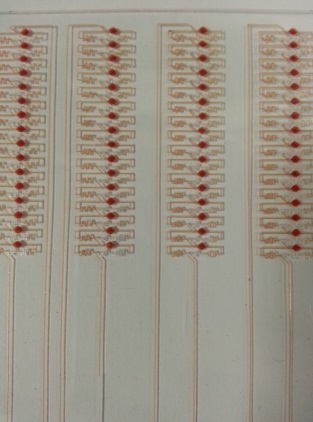

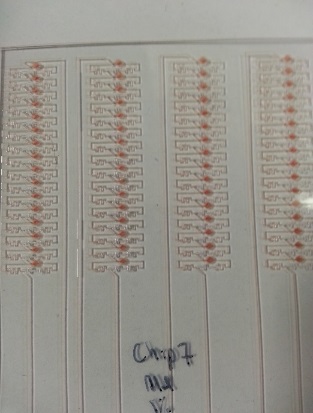

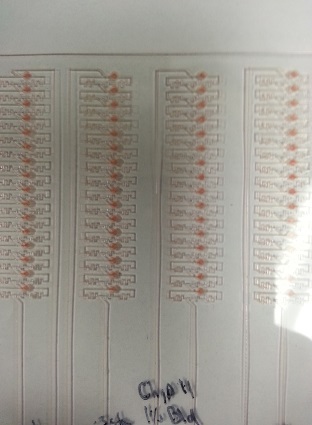

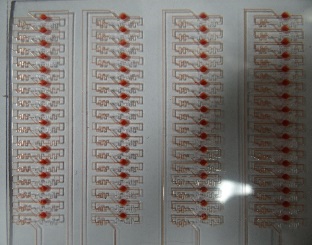

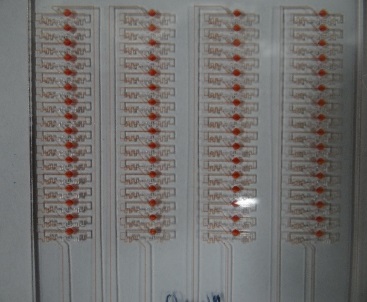


5% blood rep 1,2


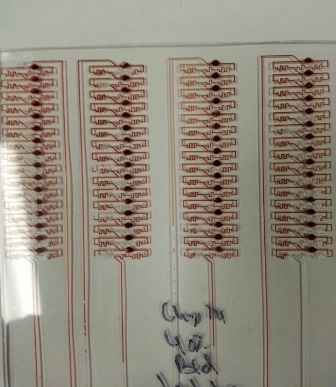


40% blood rep 1,2


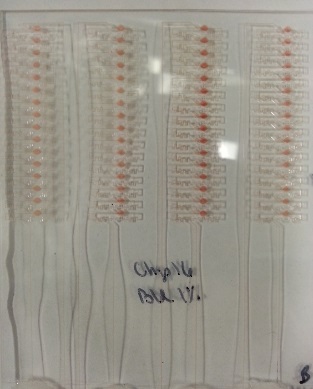


1% blood rep 1,2,3


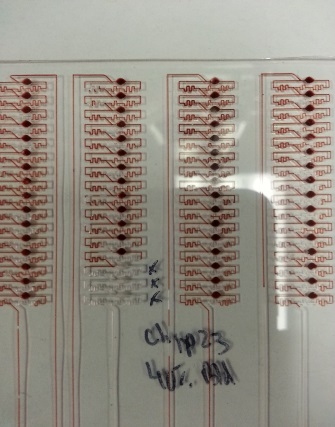


10% blood rep 1,2,3

**Fig S1.** Pictures of cards loaded with varying concentrations of blood in the final amplification reactions. Wells that did not load properly are marked with a yellow circle. Overall, 635 out of 640 wells loaded properly when tested with blood samples.
