# Supplementary material for: Static self-directed sample dispensing into a series of reaction wells on a microfluidic card for parallel genetic detection of microbial pathogens
Source: Biomed Microdevices. 2015 Aug 11;17(5):89. doi: 10.1007/s10544-015-9994-1 (PMC4531140; doi:10.1007/s10544-015-9994-1)
Supplement: Supplementary file 6 — (DOCX 534 kb) [file 10544_2015_9994_MOESM6_ESM.docx]

**
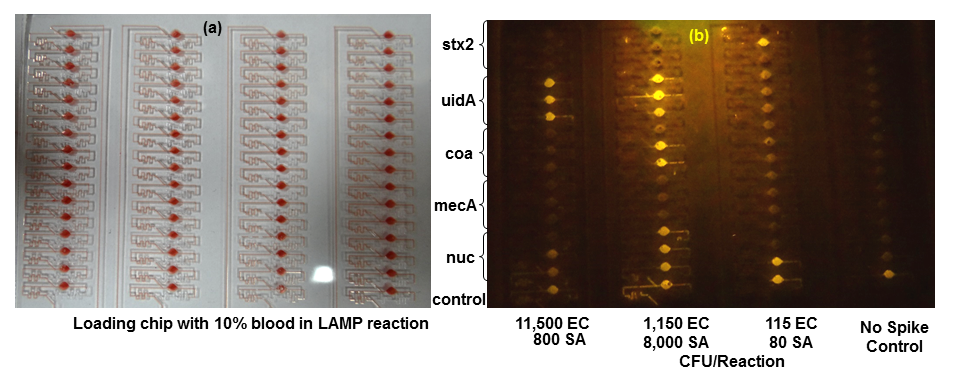
**

**Fig S2.** Card tested with spiked blood samples. Picture (captured with digital camera) of card loaded with 10% blood spiked with varying dilutions of EC (*E. coli*) and SA (*S. aureus*) CFU after a 60 min reaction time (numbers below each column indicate CFU per reaction). Each column of 16 wells was loaded with one of three serial dilutions. The fourth column was loaded with a non-spiked blood sample to serve as a negative control.
