# Supplementary material for: Static self-directed sample dispensing into a series of reaction wells on a microfluidic card for parallel genetic detection of microbial pathogens
Source: Biomed Microdevices. 2015 Aug 11;17(5):89. doi: 10.1007/s10544-015-9994-1 (PMC4531140; doi:10.1007/s10544-015-9994-1)
Supplement: Supplementary file 7 — (DOCX 836 kb) [file 10544_2015_9994_MOESM7_ESM.docx]

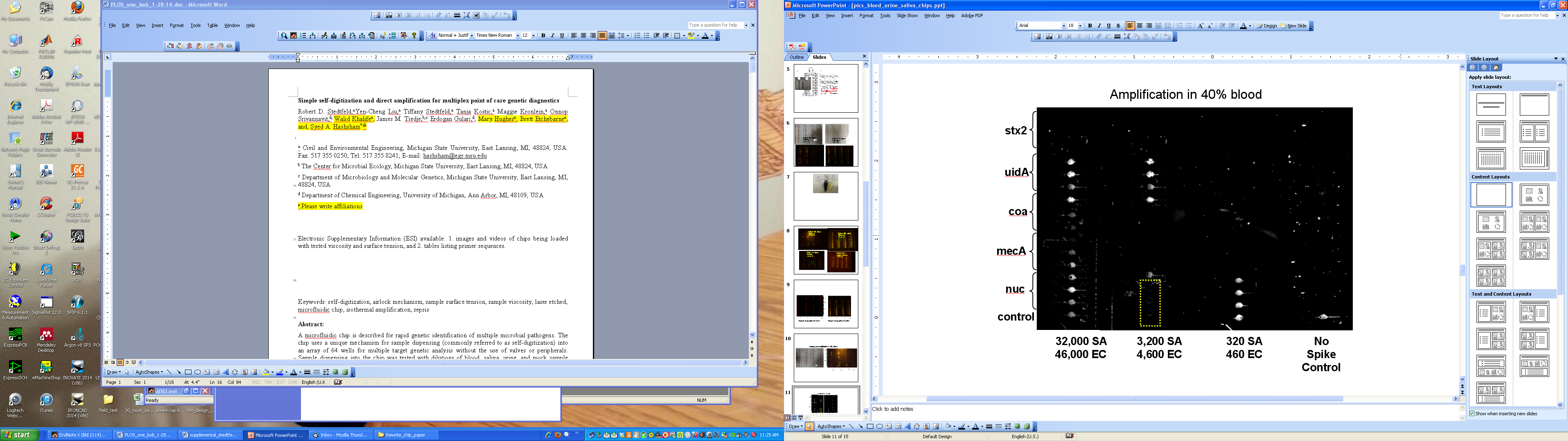


**CFU/RXN**

**Fig S3.** Card loaded with sample consisting of blood as 40% of amplification reaction, image after a 90 min incubation. Image was taken using a high exposure camera. Wells marked in yellow dashed lines did not fill properly with sample. Each column of 16 wells was loaded with a different dilution of SA (*S. aureus)* and EC (*E. coli*) CFU, listed below each column. The column on the right was loaded with a non-spiked control.
