# Supplementary material for: Static self-directed sample dispensing into a series of reaction wells on a microfluidic card for parallel genetic detection of microbial pathogens
Source: Biomed Microdevices. 2015 Aug 11;17(5):89. doi: 10.1007/s10544-015-9994-1 (PMC4531140; doi:10.1007/s10544-015-9994-1)
Supplement: Supplementary file 8 — (DOCX 347 kb) [file 10544_2015_9994_MOESM8_ESM.docx]

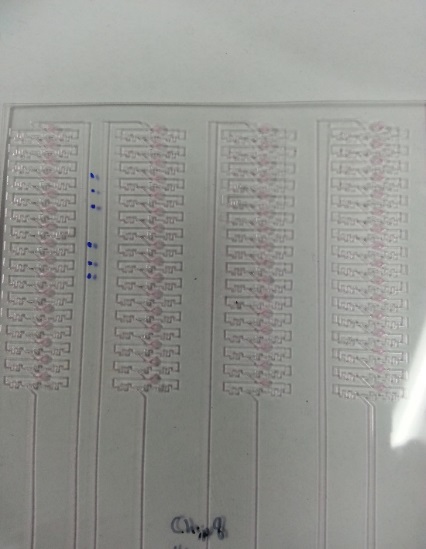

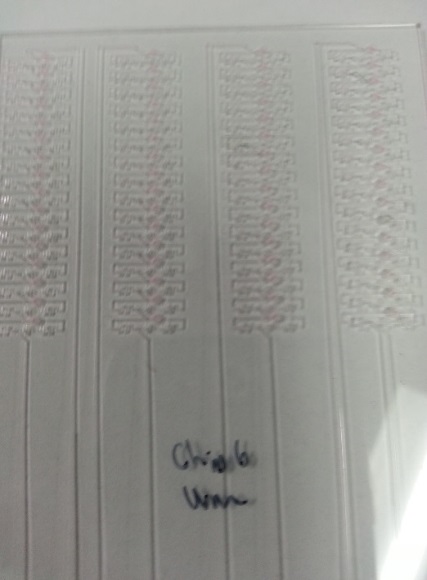

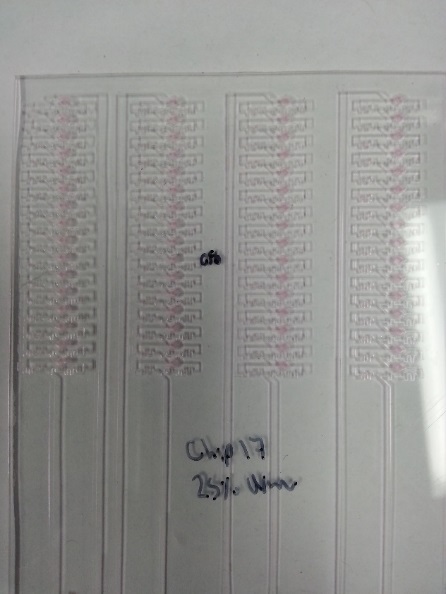

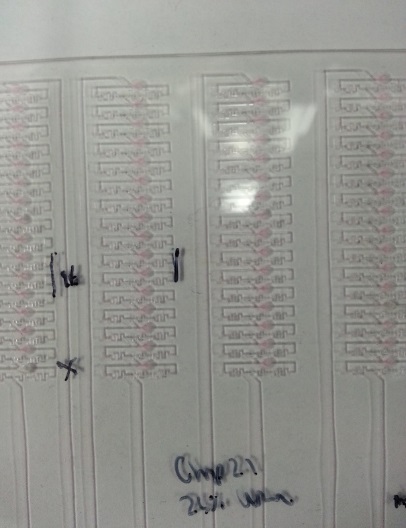

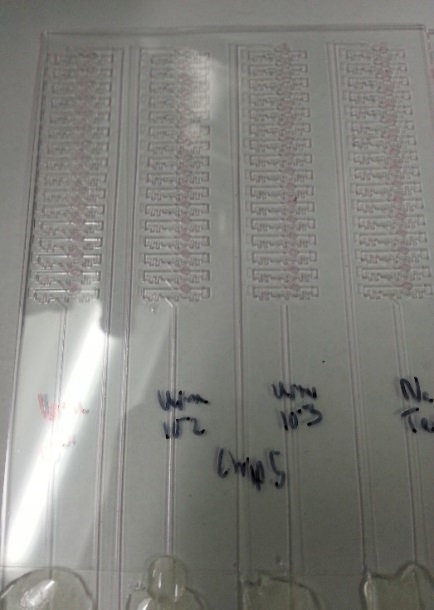


10% urine rep 1,2,3


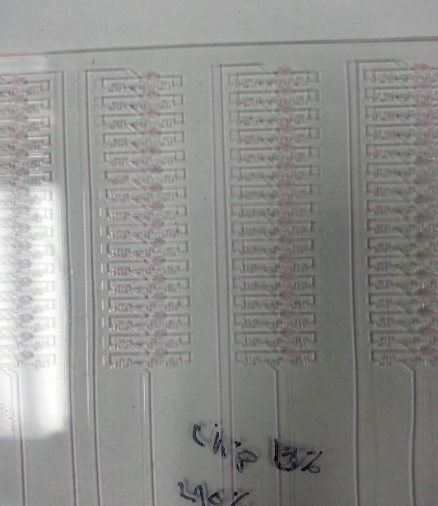


40% urine

25% urine rep 1,2

**Fig S4.** Pictures of cards loaded with varying concentrations of urine in the amplification reactions. Wells that did not load properly are marked with a yellow circle. Overall, 382 out of 384 wells loaded properly when tested with urine samples.
