# Supplementary material for: Static self-directed sample dispensing into a series of reaction wells on a microfluidic card for parallel genetic detection of microbial pathogens
Source: Biomed Microdevices. 2015 Aug 11;17(5):89. doi: 10.1007/s10544-015-9994-1 (PMC4531140; doi:10.1007/s10544-015-9994-1)
Supplement: Supplementary file 9 — (DOCX 462 kb) [file 10544_2015_9994_MOESM9_ESM.docx]

**
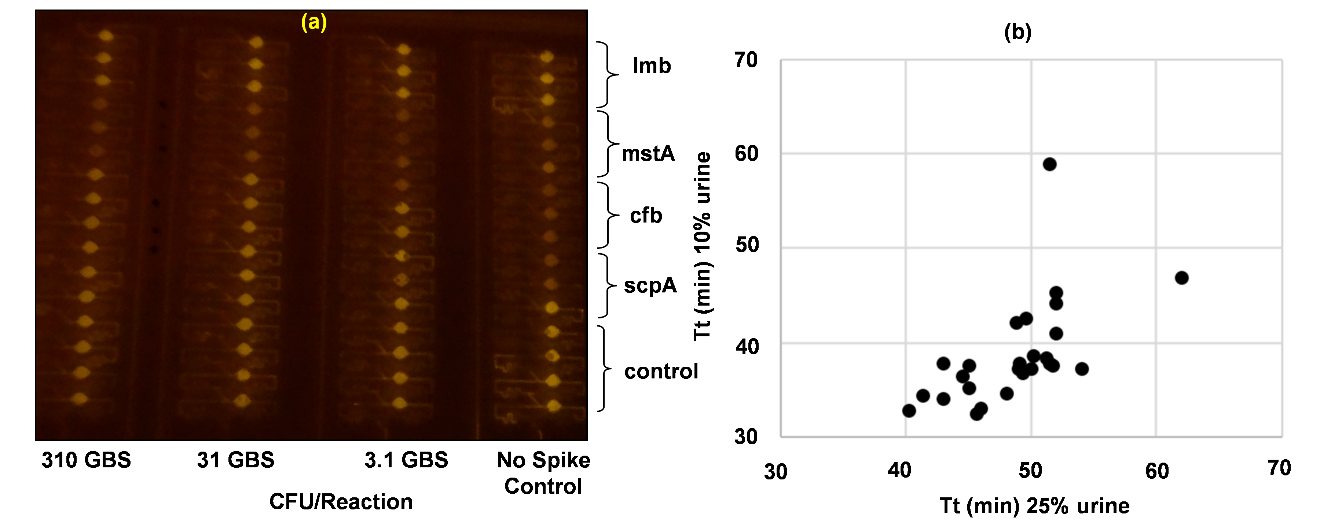
**

**Fig S5.** Card tested with spiked urine samples. (a) Picture (captured with cell-phone camera) of LAMP reaction mixed with 10% urine after 60 min incubation. Urine samples were spiked with a dilution series of GBS isolates, and each column of 16 wells was loaded with a different dilution. The fourth column was loaded with a non-spiked urine sample to serve as a negative control. The lower 4 wells were preloaded with positive control primers (cont).
