# Supplementary material for: Static self-directed sample dispensing into a series of reaction wells on a microfluidic card for parallel genetic detection of microbial pathogens
Source: Biomed Microdevices. 2015 Aug 11;17(5):89. doi: 10.1007/s10544-015-9994-1 (PMC4531140; doi:10.1007/s10544-015-9994-1)
Supplement: Supplementary file 10 — (DOCX 396 kb) [file 10544_2015_9994_MOESM10_ESM.docx]

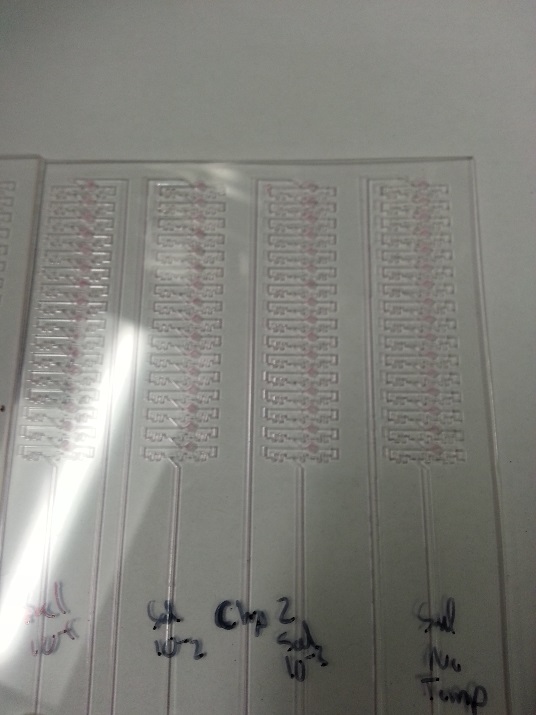

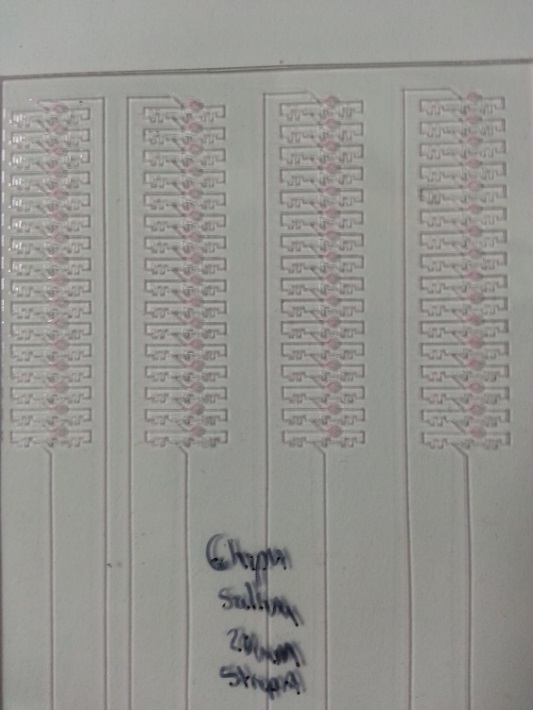

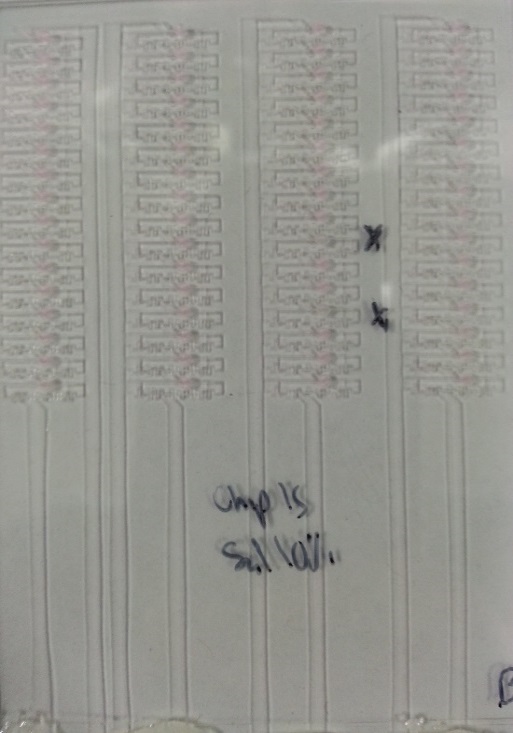

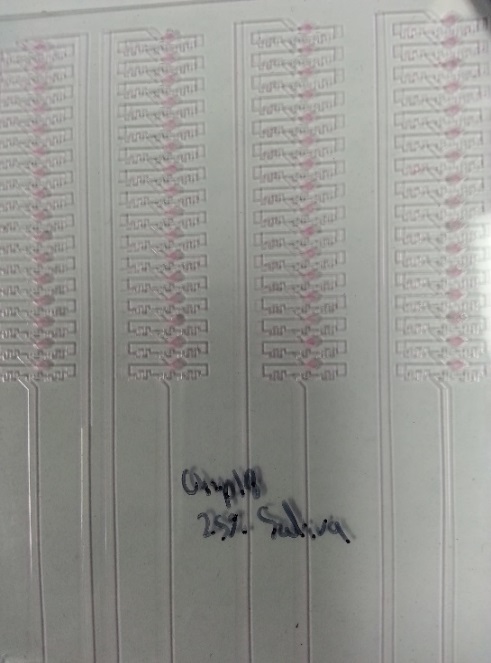

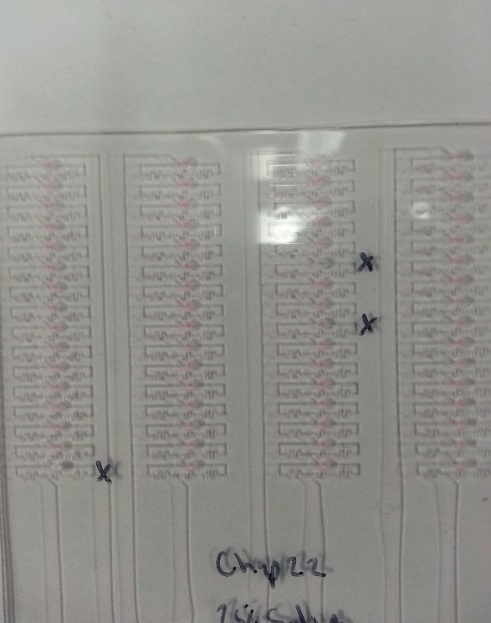


10% saliva rep 1,2,3

25% saliva rep 1,2

**Fig S6.** Pictures of cards loaded with varying concentrations of saliva in the amplification reaction. Wells that did not load properly are marked with a yellow circle. Overall, 315 out of 320 wells loaded properly when tested with saliva samples.
