# Supplementary material for: Static self-directed sample dispensing into a series of reaction wells on a microfluidic card for parallel genetic detection of microbial pathogens
Source: Biomed Microdevices. 2015 Aug 11;17(5):89. doi: 10.1007/s10544-015-9994-1 (PMC4531140; doi:10.1007/s10544-015-9994-1)
Supplement: Supplementary file 11 — (DOCX 19708 kb) [file 10544_2015_9994_MOESM11_ESM.docx]

**Fig S7.** Images of the 64-well airlock card loaded with different viscosity samples taken by a digital camera. (a) μ= 0.69 cP (b) μ= 0.89 cP (c)μ= 1.0 cP (d) μ= 1.40 cP (e) μ= 1.80cP. The dashed line shows a partial enlargement of the seventh well in the third column on each card.
