# Supplementary material for: Static self-directed sample dispensing into a series of reaction wells on a microfluidic card for parallel genetic detection of microbial pathogens
Source: Biomed Microdevices. 2015 Aug 11;17(5):89. doi: 10.1007/s10544-015-9994-1 (PMC4531140; doi:10.1007/s10544-015-9994-1)
Supplement: Supplementary file 12 — (DOCX 11200 kb) [file 10544_2015_9994_MOESM12_ESM.docx]

**Fig S8.** Result of the surface tension test for the airlock card. The three images are the 64-well airlock card loaded with samples prepared with varying surface tension (a) γ= 50 mN/m (b) γ= 60 mN/m (c) γ= 70 mN/m, respectively. The dashed line shows a partial enlargement of the seventh well in the third column on each card.
